# Supplementary material for: Evaluating the risk of comorbidity onset in elderly patients after a cancer diagnosis
Source: BMC Public Health. 2025 Feb 17;25:640. doi: 10.1186/s12889-025-21784-7 (PMC11834647; doi:10.1186/s12889-025-21784-7)
Supplement: Supplementary file 1 — Supplementary Material 1 [file 12889_2025_21784_MOESM1_ESM.docx]

**Supplementary Materials**

Supplementary Table 1: Bivariate Distribution between development of cancer vs other covariates

| **Characteristic** |  | **Cancer** | |  |
| --- | --- | --- | --- | --- |
|  | **Overall N = 6,651** | **No  N = 6,142** | **Yes N = 509** | **p-value** |
| **Gender** |  |  |  | 0.073 |
| Male (1) | 2,759.0 (41.5%) | 2,567.0 (41.8%) | 192.0 (37.7%) |  |
| Female (2) | 3,892.0 (58.5%) | 3,575.0 (58.2%) | 317.0 (62.3%) |  |
| **Race-masked** |  |  |  | <0.001 |
| White/Caucasian (1) | 5,248.0 (78.9%) | 4,800.0 (78.2%) | 448.0 (88.0%) |  |
| Black/African American (2) | 878.0 (13.2%) | 833.0 (13.6%) | 45.0 (8.8%) |  |
| Other (3) | 525.0 (7.9%) | 509.0 (8.3%) | 16.0 (3.1%) |  |
| **Hispanic** |  |  |  | 0.002 |
| No (0) | 5,845.0 (87.9%) | 5,376.0 (87.5%) | 469.0 (92.1%) |  |
| Yes (1) | 806.0 (12.1%) | 766.0 (12.5%) | 40.0 (7.9%) |  |
| **Highest degree** |  |  |  | 0.600 |
| Below College Degree (0) | 4,426.0 (66.5%) | 4,092.0 (66.6%) | 334.0 (65.6%) |  |
| College Degree and Above (1) | 2,225.0 (33.5%) | 2,050.0 (33.4%) | 175.0 (34.4%) |  |
| **BMI Proxy** |  |  |  | 0.035 |
| Normal | 2,501.0 (37.6%) | 2,291.0 (37.3%) | 210.0 (41.3%) |  |
| Obese | 1,443.0 (21.7%) | 1,355.0 (22.1%) | 88.0 (17.3%) |  |
| Overweight | 2,645.0 (39.8%) | 2,441.0 (39.7%) | 204.0 (40.1%) |  |
| Underweight | 62.0 (0.9%) | 55.0 (0.9%) | 7.0 (1.4%) |  |
| **Whether in Poverty** |  |  |  | 0.012 |
| Above Poverty (0) | 5,697.0 (85.7%) | 5,242.0 (85.3%) | 455.0 (89.4%) |  |
| Below Poverty (1) | 954.0 (14.3%) | 900.0 (14.7%) | 54.0 (10.6%) |  |
| **Smoke Ever** |  |  |  | 0.800 |
| No (0) | 3,110.0 (46.8%) | 2,875.0 (46.8%) | 235.0 (46.2%) |  |
| Yes (1) | 3,541.0 (53.2%) | 3,267.0 (53.2%) | 274.0 (53.8%) |  |

Supplementary Table 2: Unadjusted and Adjusted effects of the explanatory variables on the development of comorbidity using logistic regression model

| **Analysis of Maximum Likelihood Estimates** | | | | |
| --- | --- | --- | --- | --- |
| **Parameter** | **Unadjusted Model** | | **Adjusted** | |
|  | **Odds Ratio (95% Confidence Interval)** | **p-value** | **Odds Ratio (95% Confidence Interval)** | **p-value** |
| **Cancer (Yes)** | 1.287(1.038,1.595) | 0.022 | 1.321 (1.087,1.605) | 0.005 |
| Gender (Female) |  |  | 1.08 (0.966,1.208) | 0.177 |
| Race (White/Caucasian) |  |  | 1.025 (0.822,1.277) | 0.828 |
| Race (Black/African American) |  |  | 1.068 (0.821,1.39) | 0.624 |
| Hispanic (Yes) |  |  | 0.991 (0.824,1.193) | 0.927 |
| **Highest degree (College Degree and Aboce)** |  |  | 0.792 (0.702,0.893) | <0.001 |
| **BMI (Obese)** |  |  | 1.803 (1.561,2.082) | <0.001 |
| BMI (Overweight) |  |  | 1.268 (1.117,1.439) | <0.001 |
| BMI (Underweight) |  |  | 1.387 (0.805,2.39) | 0.239 |
| Whether in Poverty (Below Poverty) |  |  | 1.223 (1.045,1.431) | 0.012 |
| **Smoke ever (Yes)** |  |  | 1.264 (1.132,1.412) | <0.001 |
